# Acute ethanol exposure during late mouse neurodevelopment results in long‐term deficits in memory retrieval, but not in social responsiveness

**DOI:** 10.1002/brb3.636

**Published:** 2017-03-21

**Authors:** Katherine Houlé, Myshake Abdi, Erin B. D. Clabough

**Affiliations:** ^1^ Division of Pulmonary and Critical Care MedicineMedical College of South CarolinaCharlestonSCUSA; ^2^ Department of BiologyRandolph‐Macon CollegeAshlandVAUSA; ^3^ Department of BiologyHampden‐Sydney CollegeFarmvilleVAUSA

**Keywords:** adult behavior, binge drinking, development, ethanol, fetal alcohol spectrum disorders, learning/memory, mouse model

## Abstract

**Objective:**

Prenatal alcohol exposure can result in neurological changes in affected individuals and may result in the emergence of a broad spectrum of neurobehavioral abnormalities termed fetal alcohol spectrum disorders (FASD). The effects of ethanol exposure during development are both time and dose dependent. Although many animal models of FASD use more chronic ethanol exposure, acute developmental alcohol exposure may also cause long‐lasting neuronal changes. Our research employed behavioral measures to assess the effects of a single early postnatal ethanol intoxication event in mice.

**Materials and Methods:**

Mice were dosed at postnatal day 6 (a 2.5 g/kg dose of ethanol or a saline control administered twice, 2 hr apart) as a model of third trimester binge drinking in humans. This exposure was followed by behavioral assessment in male mice at 1 month (1M) and at 4 months of age (4M), using the Barnes maze (for learning/memory retrieval), exploratory behavior, and a social responsiveness task.

**Results:**

Ethanol‐exposed mice appeared to be less motivated to complete the Barnes maze at 1M, but were able to successfully learn the maze. However, deficits in long‐term spatial memory retrieval were observed in ethanol‐exposed mice when the Barnes maze recall was measured at 4M. No significant differences were found in open field behavior or social responsiveness at 1M or 4M of age.

**Conclusions:**

Acute ethanol exposure at P6 in mice leads to mild but long‐lasting deficits in long‐term spatial memory. Results suggest that even brief acute exposure to high ethanol levels during the third trimester equivalent of human pregnancy may have a permanent negative impact on the neurological functioning of the offspring.

## 1 Introduction

Alcohol, a known physical and behavioral teratogenic drug, causes debilitating disruptions in neurodevelopment (Sampson et al., 1997). Effects of ethanol on neurodevelopment are dependent on the timing and duration of exposure. Prenatal exposure to alcohol can cause structural abnormalities to multiple brain regions that result in a broad spectrum of neurobehavioral and cognitive abnormalities classified as fetal alcohol spectrum disorders (FASD; Mattson & Riley, 1998).

Researchers investigating FASD frequently use animal models due to the ability to control the timing and amount of fetal and postnatal ethanol exposure (Hunt & Barnet, 2015). In particular, rodent models of FASD are commonly used. Though there are a vast number of different rodent FASD models, researchers have been able to characterize temporal benchmarks in rodent neurodevelopment and behavior that correlate with the human condition (Semple, Blomgren, Gimlin, Ferriero, & Noble‐Haeusslein, 2013). These neurodevelopmental benchmarks allow for researchers to use varying ethanol treatment paradigms as a relatively faithful mimic of the human condition.

Chronic mouse models of FASD have shown that prenatal ethanol exposure can alter motor functioning (Ornelas, Novier, Van Skike, Diaz‐Granados, & Matthews, 2015), learning and memory (Marquardt & Brigman, 2016), and social behaviors (Varlinskaya & Mooney, 2014). However, fewer research studies have used an acute ethanol exposure model to study behavior, so effects of acute ethanol exposure therefore remain somewhat less certain. Acute ethanol exposure can allow the researcher to identify more subtle behavioral changes that may occur in the absence of chronic exposure, as well as target more specific developmental periods, such as neurulation, neuronal migration, or synaptogenesis.

Acute ethanol exposure (1 g/kg for 1 hr/day over 3 days) in late gestation causes fetal white matter depletion that could hinder brain connectivity and function in sheep (Dalitz, Cock, Harding, & Rees, 2008). In mice, a single‐day binge ethanol exposure during late brain development creates apoptotic neurodegeneration within 24 hr in brain regions that include specific areas of the cortex (frontal, cingulate, parietal, temporal, and retrosplenial cortex), as well as the hippocampal formation (CA1 and subiculum), striatum, anterior thalamus, and mammillary bodies (Ikonomidou et al., 2000; Olney et al., 2002; Saito, Chakraborty, Mao, Paik, & Vadasz, 2010; Wilson, Peterson, Basavaraj, & Saito, 2011; Wozniak et al., 2004).

**Figure 1:**
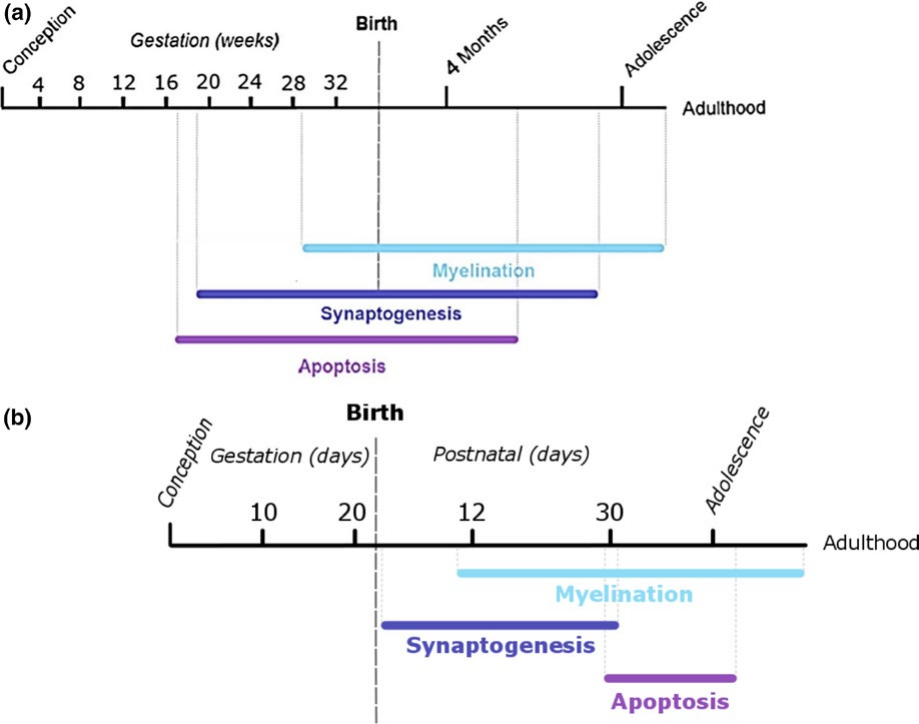
A timeline chronologically displaying the sequential and sometimes overlapping major stages of late brain development, including synaptogenesis, myelination, and apoptosis in both humans (a) and mice (b). Adapted from Tau and Peterson (2010) and Semple et al. (2013)

We chose to look at the effects of ethanol exposure during the period of neurodevelopment that encompasses synaptogenesis. This period occurs predominantly prenatally in humans (Figure 1a) and postnatally in mice (Figure 1b; Ikonomidou et al., 2000). The neurodevelopment that occurs between human gestational months 7 and 9 is roughly equivalent to the processes that occur in mice and rats from birth until postnatal 2 weeks (Susick, Lowing, Provenzano, Hildebrandt, & Conti, 2014). In particular, the time period between embryonic day 19 and postnatal day 14 in mice is most sensitive to neurodegeneration due to ethanol exposure (Ikonomidou et al., 2000), likely due to simultaneous ongoing synaptogenesis.

In the current study, we sought to measure the short‐ and long‐term behavioral responses of mice to a single acute intoxication event (a 2.5 g/kg dose of ethanol or a saline control administered twice, 2 hr apart) as a model of third trimester drinking in humans (Olney et al., 2002). Our investigations describe the effect of this ethanol exposure at postnatal day 6 (P6) on short‐ and long‐term memory through the ability to learn a maze and retain that information, as well as explore the impact of ethanol exposure on general locomotor activity, exploratory behavior, and social responsiveness to a trapped conspecific.

## 2 Methods

### 2.1 Animals

Protocols for animal use were approved by the Institutional Animal Care and Use Committee at Randolph‐Macon College. All mice were offspring from timed pregnant C57BL6/J females (Jackson Laboratories), received on the same gestational day and subsequently housed in a temperature‐controlled room under 12‐hr light/dark cycles (lights off at 6 p.m.), with ad libitum access to food and water. Behavioral testing was done at the end of the light cycle. Each litter was split into treatment levels by random assignment. Both genders in the six litters were pooled together for early weight measurements, whereas only male mice were used in the adult behavioral analyses.

Mice pups were weaned and males were housed singly beginning at P32. Some litters did not have multiple male pups, and in order to avoid fighting that can occur when nonlitter mate males are cohoused, single‐male housing was employed. Though social isolation may be considered a stressor that may induce changes in mice behavior and anxiety coping, other researchers have found that group‐housed mice displayed more anxious behaviors than socially isolated mice prior to behavioral testing (Lopez & Laber, 2015).

### 2.2 Injections

Saline and ethanol solutions were made with normal saline solution (NaCl) acting as a control (Spong, Abebe, Gozes, Brenneman, & Hill, 2001). The ethanol solution was a 2.5 g/kg dose, which was determined using the density of ethanol C_2_H_5_OH as 0.78929 g/ml. The ethanol solution was made with a mixture of 100% ethanol and the control saline solution, which rendered the proper concentration of a 20% ethanol solution. At postnatal day 6 (P6), pups were injected subcutaneously at a 20‐degree angle near the back of the neck, using a 100 μl Hamilton syringe with a 1 inch 28‐gauge needle point. The ears of the mice pups were clipped in order to distinguish between the treatment levels, with right ear clipped for saline injection, and left ear clipped for ethanol injection. The pups were marked with ink and immediately returned to the mother in the home cage following injection. Mice were injected with a second 2.5 g/kg ethanol dose or saline control 2 hr following the first injection.

Multiple studies have shown that C57BL/6 mice are quite sensitive to early postnatal EtOH treatment and the early postnatal injection paradigm used in our study has been well‐characterized: C57BL/6J pups treated with a single 2.5 g/kg dose of ethanol between P5–7 reach a blood ethanol content (BEC) of ~250 mg/dl approximately 45 min after injection (Susick et al., 2014) If the dose is then repeated subcutaneously after 2 hr, as in our paradigm, C57BL/6 pups achieve a mean BEC 1 hr after the second injection of 472 (±16) mg/dl, with an alcohol clearance rate of 283 mg dl^−1^ hr^−1^ (Wagner et al. 2014).

Similarly, a separate study showed P7 ethanol treatment induces a peak blood alcohol level of 0.5 g/dl when truncal blood was collected at 0.5, 1, 3, and 6 hr following the second ethanol injection (Saito et al., 2007). Specifically, BEC spectrophotometer readings of absorbance of fluorescence at 340 nm in C57BL/6 pups show two peaks: The first peak (270 mg/dl) occurs 45 min after the first 2.5 g/kg dose, while the second peak (510 mg/dl) occurs 1 hr after the second 2.5 g/kg dose (3 hr after the first dose; Wozniak et al., 2004).

Based on this data, our acute single intoxication paradigm maintains a toxic blood ethanol concentration above 200 mg/dl for several hours, which is the minimum level needed for triggering neurodegeneration consistently (Carloni, Mazzoni, & Balduini, 2004; Olney et al., 2002). This blood ethanol level is in the range that a human fetus might be exposed to after maternal ingestion of a moderate to heavy dose of ethanol (Ikonomidou et al., 2001).

### 2.3 Weights and righting

The weights of all pups were taken before injection at P6 and used to calculate the amount of solution to inject into each specific mouse pup. There was no significant difference in the P6 preinjection weights between treatment groups (by *t* test, *p* = .984, mean of saline mice = 3.26 g ± 0.08 *SE*, mean of ethanol mice = 3.26 ± 0.10 *SE*).). At 24 hr postinjection (P7), all pups were reweighed and a behavioral righting measure was performed to obtain information on motor coordination. The time it took each mouse to flip onto all four paws from laying on their backs was recorded, as a modified version of a protocol performed by Palanza, Howdeshell, Parmigiani, & Vom Saal, 2002 (*n* = 19 ethanol‐injected mice and 17 saline‐injected mice).

In addition, weights were taken at P30 for males and females (*n* = 9 ethanol‐injected and 7 saline‐injected male mice; *n* = 5 ethanol‐injected and 5 saline‐injected female mice), and again at 4M of age for males (*n* = 8 ethanol‐injected male mice and *n* = 6 saline‐injected male mice).

### 2.4 Construction of Barnes maze

The Barnes maze measures the mouse's ability to learn the location of a target zone with the use of visual cues, and is a measure of spatial learning and memory (Harrison, Hosseini, & McDonald, 2009). A traditional 20‐hole circular Barnes maze measuring 120 cm in diameter was constructed from wood, painted glossy white, and positioned at a height of 120 cm above the floor. Each hole measured 4.5 cm in diameter. The holes were evenly spaced on the perimeter of the maze, located 2.5 cm away from the maze edge and 13 cm away from neighboring holes. Black discs were secured under each hole and a 23 × 11 cm black box was attached under the specified target hole as an escape tunnel. Large symbols (a triangle, circle, and cross) were placed on the walls as visual cues for the mice.

### 2.5 Barnes maze procedure

Mice were trained on the Barnes maze beginning at P32 for seven trials, and followed by a single session long‐term trial at 4M. Only male mice were run on the Barnes maze (ethanol *n* = 8, saline *n* = 6). During training, mice were run on the maze daily for 7 days during the early afternoon. Each mouse ran the maze only once per day, for a total of seven exposures to the maze.

A 100 W light was placed 25 cm over the center of the platform. To ensure the mice were properly motivated to enter the target hole, an additional LED light and an ultrasonic noisemaker (1.9 W) were also hung 25 cm above the center of the maze on trial day 5. The ultrasonic noise was turned on when each mouse was placed in the center of the maze, and then turned off as soon as the mouse entered the target hole.

Each mouse was randomly assigned a unique target hole, and these targets stayed constant for each mouse throughout the duration of the measure. All of the mice were housed in separate cages, and the cages were kept outside of the testing room. Each mouse began a trial facing the same direction and was allowed to explore the maze for a maximum of 5 min. If the mouse did not enter the target box within 5 min, it was corralled into its respective target hole, and a cover was placed over the target hole to ensure that the mouse was unable to climb out of the target. Following entry into the target hole, the mouse was allowed to rest in the dark target box for 1 min, and the ultrasonic noisemaker was turned off during this time (trials 5–7). After each trial, the mouse was returned to an individual cage, and the maze and target box were cleaned with 70% ethanol.

During each trial, the researcher recorded the movements of the mouse, including the latency to reach the first hole, the latency to find the target hole, the number of errors before finding the target, the first hole's distance from the target, and the latency to enter the target. The data for trials 1–7 were analyzed, using one‐way repeated measures ANOVA.

The 4M single long‐term probe trial was run under the same conditions as the P32 trials (using the same matched target box for each mouse). The same data were collected, but in addition, the percent of the holes explored on the exact opposite quadrant of the maze was also recorded. Search strategy was also analyzed. Exploration of the opposite quadrant of the maze could indicate a failure to remember the portion of the maze containing the target escape hole. Data were analyzed by independent *t* tests at 4M.

### 2.6 Exploratory behavior measure

Open field assays measure locomotor activities, but can also be used to assess ability to cope with the stress‐inducing aspects of a novel environment (Mothes, Opitz, Werner, & Clausing, 1996; Prut & Belzung, 2003). To assess the impact of developmental ethanol exposure on locomotion and exploratory behavior in a novel environment, male mice were exposed to a novel arena at P30 and 4M. The arena was a 38 × 38 cm square plastic box with raised walls of 19 cm, filled with corn bedding to a height of approximately 2.5 cm (Prut & Belzung, 2003). A clear empty cylindrical chamber (measuring 4 cm L; 3 cm circumference) was placed in the center of the arena on its side.

Each male mouse was placed in the arena facing the lower left corner of the box and allowed to explore the arena for 10 min, followed by removal from the arena. The space was divided into nine equal sections for analysis, with the center section containing the empty cylinder. The number of quadrants crossed, the time spent in the center of the arena, the number of digging and grooming episodes, and the number of time the mice touched the cylindrical center chamber were recorded. The first 5 min of exploratory behavior was used to assess locomotor behavior (by the number of quadrants crossed by the mice) and exploratory behavior (by the time spent in the center section of the arena near the chamber). The data were analyzed by independent *t* tests.

### 2.7 Social responsiveness measure (damsel in distress)

In order to determine if a single intoxication event has an impact on social behavior, male mice were exposed to a social responsiveness paradigm at both P30 and 4M, using the above described arena. In this social responsiveness measure, a female mouse was placed in the previously empty central container. The chamber was small enough so that the female could not turn around inside of the tube and was trapped in a forward facing position.

Following the 10‐minute male exploration of the empty arena at P30, the male was removed from the field. A female littermate was placed into the small chamber and allowed to acclimate for 5 min, after which time the male littermate was placed back into the arena. The researcher documented the response of the littermate male to the presence of the trapped female by recording the number of quadrants crossed, the time spent in the center of the arena where the chamber was located, the number of digging and grooming episodes in the center and in other quadrants, the number of times the mouse touched the chamber, and the number of times noses were touched between the male and trapped female.

After the measure was completed, both mice were taken out of the arena and placed into solitary holding containers. The arena and tube were cleaned with 70% ethanol, and the corn bedding was replaced after the completion of every trial. At 4M of age the same measure was repeated, using nonlittermate females, as some litters did not have a surviving female at 4M and researchers wanted to avoid bias in the collected data. The central chamber used was a slightly larger to accommodate for the larger body size of the mice at the 4M time point, but the female mice were still unable to turn around inside of the chamber. At both time points, the data was analyzed by independent *t* tests, with the time spent in the center of the arena near the chamber and touching the chamber as the main measure of social responsiveness.

## 3 Results

### 3.1 Weights

**Figure 2:**
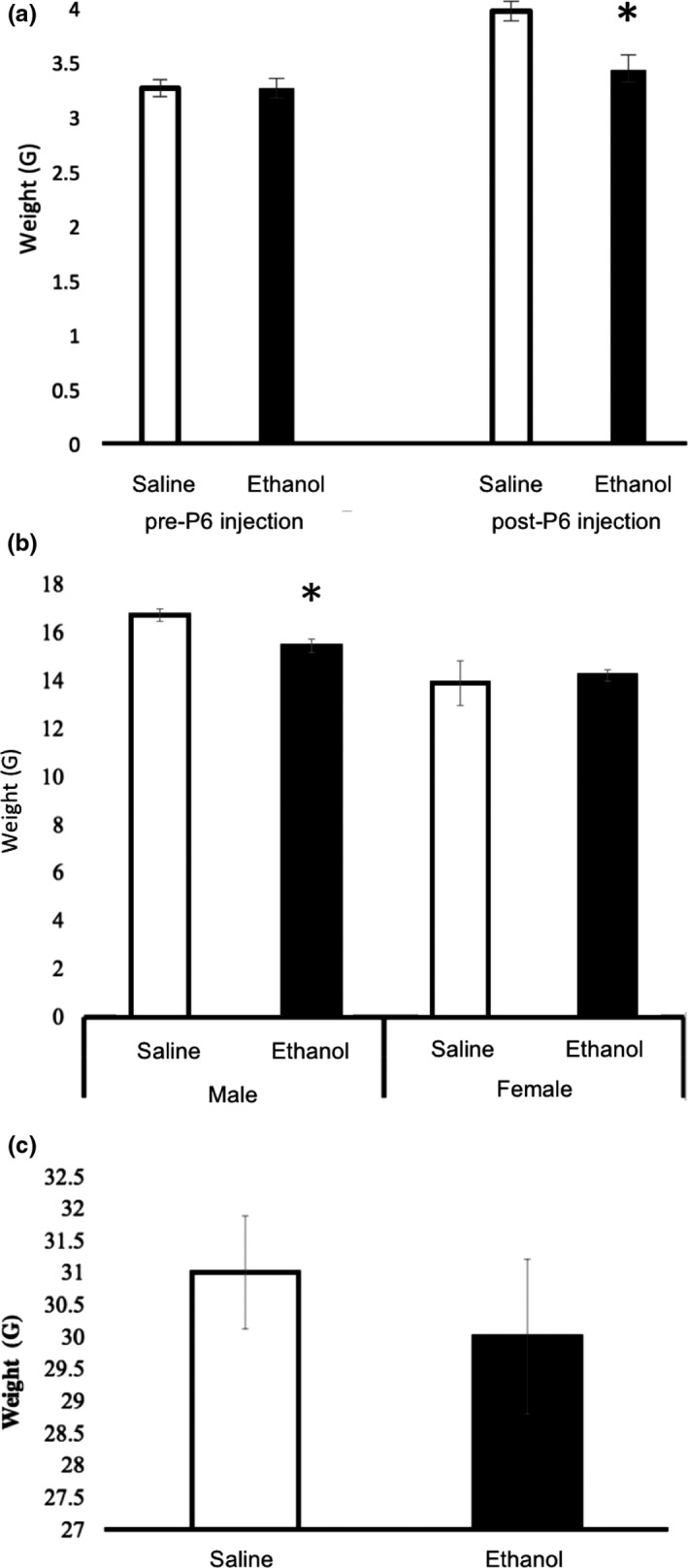
Measured weights of P6 ethanol‐exposed mice compared to saline control at P7, P30, and 4 months. (a) Ethanol‐injected mice pups (*n* = 19) weighed significantly less than saline‐injected pups (*n* = 17) at P7 (24 hr after injection). P7 data were pooled gender (by *t* test, *p* = .0016). (b) Mean P30 weights of male (ethanol *n* = 9, saline *n* = 7) and female (ethanol *n* = 5, saline *n* = 5) mice pups injected at P6 with ethanol or saline (by *t* test, male *p* = .0076, female *p* = .7785). (c) Male mice showed no significant difference in weight by 4 months post‐P6 ethanol (*n* = 8) or saline (*n* = 6) injection (by *t* test, *p* = .5465). Error bars indicate *SEM*; * indicates significance at *P* < 0.05

Mouse pups injected with ethanol at postnatal day 6 (P6) weighed significantly less than pups injected with saline at 24 hr after injection on P7 (pooled gender; by *t* test; *p* = .0017; Figure 2a). There was no significant difference in righting reflex measurements taken 24 hr after injection with ethanol or saline (pooled gender; by *t* test; *p* = .2309). Ethanol‐exposed male pups still weighed significantly less than saline‐injected male pups at P30 (by *t* by test; *p* = .0076, Figure 2b). However, ethanol status made no difference in weights for female mice pups at p30 (by *t* test; *p* = .7785). By 4M, the acute ethanol exposure showed no effect on the weights of male mice (by *t* test; *p* = .5465, Figure 2c).

### 3.2 Barnes maze: training

**Figure 3:**
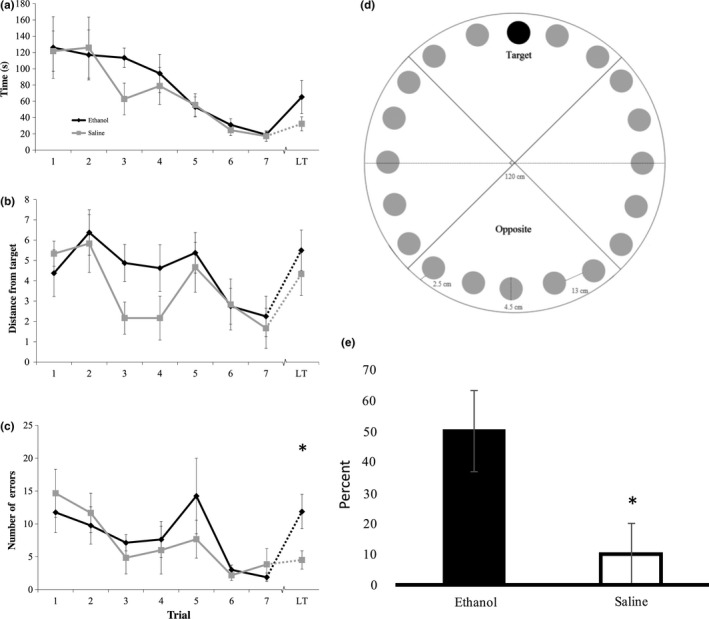
Barnes maze performance as a measure of learning and memory. P6‐ethanol exposed mice and saline mice were trained on the Barnes maze at 1 month of age. Mice learned the location of the target hole over the course of multiple sessions (trials 1–7). At 4M, mice were tested again in the long‐term (LT) session for their ability to recall the location of the target hole several months after the training. (a) There was no difference in latency to find the target between ethanol and saline‐injected mice (ethanol *n* = 8, saline *n* = 6) over the course of the seven trial days or on the LT trial (b) There was no difference in the distance between the first hole and the target during training between ethanol and saline‐injected mice during training or on the LT trial. (c) There was no difference in the number of wrong holes explored before the target during training days, but ethanol‐injected mice made significantly more errors on the LT trial before finding the target hole (by *t* test, *p* = .0311). (d) The Barnes maze was divided into four quadrants for analysis based on the target location. An increase in the exploration of the opposite quadrant from the target indicates a spatial learning deficit (Attar et al., 2013). (e) Ethanol‐injected mice explored the quadrant opposite from their target quadrant more frequently than saline‐injected mice on the LT trial (by *t* test, *p* = .032). Error bars indicate *SEM*; * indicates significance at *P* < 0.05

Both ethanol and saline‐injected mice were able to successfully learn the Barnes maze task at P32, as evidenced by a decrease in the latency to find the target over the course of seven trials regardless of treatment group (by ANOVA; *F*_1,6_ = 5.720; *p* < .001). Latency to find the target indicates how many seconds passed before the mouse located the target hole and was used as a measure of learning. Ethanol status made no difference in the speed at which the mice located the target over the course of seven trials (by ANOVA, *F*_1,6_ = 0.260; *p* = .953; Figure 3a).

Mice should be randomly exploring during the initial trial, but as learning occurs, the distance between the first hole explored and the target hole should decrease. Therefore, the distance of the first explored hole from the specified target was used as a measure of learning across the treatment levels. There was a significant difference in the first hole distance from target over the course of the seven trials regardless of treatment group (By ANOVA; *F*_1,6_ = 3.909; *p* = .002), indicating that learning is occurring in both groups. Ethanol status did not significantly impact the distance between the first hole explored and the target over the seven training trials (By ANOVA; *F*_1,6_ = 0.817; *p* = .56; Figure 3b), nor did it affect the overall number of errors that the mice made during the training trials (Figure 3c).

### 3.3 Barnes maze: long‐term trial

**Figure 4:**
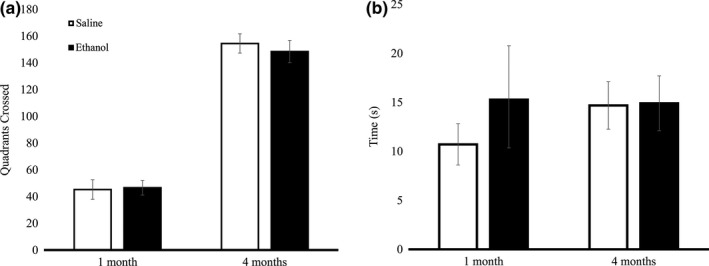
P6 ethanol‐injected mice did not differ from saline‐injected mice in exploratory behavior in the open field locomotion assay. (a) Mean number of quadrants crossed by ethanol and saline mice were not significantly different at P30 (ethanol *n* = 8, saline *n* = 7; by *t* test, *p* = .443) or at 4 months (ethanol *n* = 6, saline *n* = 6; by *t* test, *p* = .298). (b) Time spent in the center of the arena without a female mouse in the chamber did not differ between ethanol and saline‐exposed mice at 1 month (ethanol *n* = 8, saline *n* = 7; by *t* test, *p* = .207) or at 4 months (ethanol *n* = 6, saline *n* = 6; by *t* test, *p* = .478). Error bars indicate *SEM*

However, several measures detected a deficit in memory retrieval seen in the long‐term trial. The long‐term trial revealed no significant differences in the latency to find the target (by *t* test, *p* = .17, mean of saline mice = 32.33 s ± 8.6 *SEM*; mean of ethanol mice = 65.25 ± 20.44 *SEM*) nor in how far the first explored hole was from the target (by *t* test, *p* = .44, mean of saline mice = 4.33 ± 1.05 *SEM*; mean of ethanol mice = 5.5 ± 1 *SEM*) when measured at 4 months post‐P6 injection and approximately 3 months after the mice learned the maze. Though no difference in error rate was detected during training, the ethanol‐injected mice made significantly more errors before finding the target than saline‐injected mice during the long‐term trial (by *t* test; *p* = .0311, mean of saline mice = 4.5 ± 1.38 *SEM*; mean of ethanol mice = 11.88 ± 2.61 *SEM*; Figure 4c).

With the Barnes maze divided into four scoring quadrants (Figure 4a), ethanol‐injected mice explored a higher percent of opposite quadrant holes than did saline‐injected mice during the long‐term trial (by *t* test; *p* = .032; Figure 4b), indicating a deficit in spatial long‐term memory retrieval. We chose to examine opposite quadrant exploration because quantifying the percentage of correct quadrant holes explored does not reflect Barnes maze learning accurately—animals with perfect performance who proceed directly to the target hole would receive a 0%, but a mouse who did not know where the target was and never searched the right quadrant would also receive a 0%. In fact, statistical analysis shows that the percentage of holes explored in the correct quadrant is significantly different between groups, but saline mice actually explored fewer holes in the correct quadrant before finding the target (by *t* test, *p* = .023, mean of saline mice = 25 ± 9.13% *SEM*, mean of ethanol mice = 53.3 ± 3.13% *SEM*). This likely reflects a more targeted approach by the saline mice, as the ethanol mice also statistically explored more holes before finding the target (termed errors).

Further analysis of search strategy revealed that all of the ethanol mice employed a “ring” search strategy during the long‐term trial (100%), where the mice started at a hole and then sequentially searched at least four holes until they encountered the target, whereas only 16.7% of the saline mice employed this strategy (by *t* test, *p* = .004).

There was a significant difference in the total number of grooming episodes over the course of the Barnes maze testing, with ethanol‐injected mice (mean = 0.767, *SEM* = 0.135) displaying more grooming behavior than saline‐injected mice (mean = 0.143, *SEM* = 0.064; by *t* test; *p* = .001). The differences in total grooming episodes were more pronounced during the first two days of Barnes maze testing (*t* test; day 1 *p* = .005, day 2 *p* = .012). Ethanol‐injected mice (day 1 mean = 1.125, *SEM* = 0.226; day 2 mean = 1.625, *SEM* = 0.375) displayed significantly more grooming behavior during the first 2 days of Barnes maze testing than saline‐injected mice (day 1 mean = 0.167, *SEM* = 0.167; day 2 mean = 0.333, *SEM* = 0.211). A post hoc power analysis revealed that on the basis of the means, the *n* values of 6 and 8 were sufficient for power at or above the recommended .8 level for the number of errors, the quadrant analysis, search strategy, and the grooming data.

### 3.4 Exploratory and social behavior

There was no significant difference in exploratory behavior between ethanol and saline mice at P30 (by *t* test, *p* = .443) or 4 months (by *t* test, *p* = .298) as measured by the open arena behavioral test (Figure 4a). There was no significant difference in time spent in the center of the arena between ethanol and saline male mice at P30 (by *t* test, *p* = .207) and 4 months (by *t* test, *p* = .478) when there was not a female mouse in the chamber (Figure 4b).

**Figure 5:**
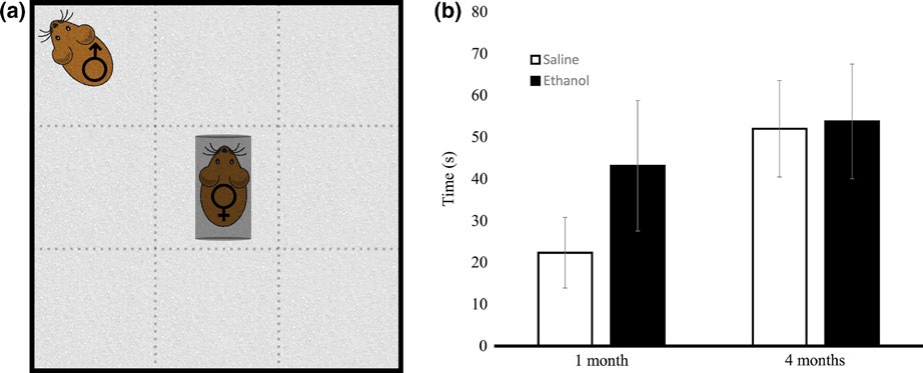
Damsel‐in‐Distress social responsiveness paradigm. Ethanol‐exposed mice did not display significant differences as compared to the saline controls when observed during the social experiments. (a) In the Damsel‐in‐Distress experiment, a female mouse was trapped in a narrow chamber at the center of the open field. A male mouse was placed in the corner of the open field and measures of social responsiveness were scored in the presence of the age‐matched female. (b) There was no difference in the time spent in the center of the arena for ethanol and saline mice at 1 month (ethanol *n* = 8, saline *n* = 7; by *t* test, *p* = .133,) or at 4 months (ethanol *n* = 6, saline *n* = 6; by *t* test, *p* = .463). Error bars indicate *SEM*

When a trapped female mouse was in the chamber in the center of the open field for the damsel‐in‐distress paradigm (Figure 5a), we saw no difference in social responsiveness between ethanol and saline‐injected male mice during the P30 (by *t* test, *p* = .133) or 4 month trial (by *t* test, *p* = .463; Figure 5b). In addition, there was no significant difference in the number of grooming episodes, number of digging episodes, or the number of times noses were touched between the male and trapped female in either group. A post hoc power analysis revealed that on the basis of the means, the *n* values were sufficient for power at or above the recommended .8 level for the damsel‐in‐distress measures.

## 4 Discussion

The present study contributes to the understanding of a single intoxication event during a developmental period analogous to the third trimester of human gestation. In mouse models, both physical and behavioral responses to acute ethanol exposure were measured. Ethanol‐injected mice pups weighed significantly less than saline‐injected pups 24 hr after injection, likely due to ethanol‐exposed pups' inability to nurse during intoxication. At P30, ethanol‐injected male mice offspring weighed significantly less than saline‐injected male pups, though this phenomenon was not found among the female offspring.

Studies have shown that exposure to high, not low, ethanol levels during a third trimester equivalent results in reduced weight at postnatal days 2–9 in rats (Puglia & Valenzuela, 2010). It is unclear why our high acute dose prevented normal weight gain at P30 in males only, but it is known that chronic ethanol exposure during the early postnatal period reduces weight gain at P30 in a manner that is more likely attributable to the effects of ethanol on fetal growth, rather than undernutrition (Lugo, Marino, Cronise, & Kelly, 2003).

Previous research shows that chronic ethanol administration can impair social recognition and behavior in adolescent rats (Kelly & Tran, 1997; Lugo et al., 2003). We did not find any significant differences in social responsiveness between ethanol and saline‐injected mice pups when tested in adolescence or adulthood. Acute administration of ethanol during various embryonic time points (E7–E15) to target specific brain region development can also induce social phenotypes (Mooney & Varlinskaya, 2011; Varlinskaya & Mooney, 2014), but our acute exposure may either be of an insufficient dose to cause marked effects on social interactions later in life, or the postnatal exposure may miss a distinct embryonic critical window for social development.

Our experiment found all mice were able to successfully learn the Barnes maze target location over a 7‐day period at 1M. However, when placed in the Barnes maze during the long‐term trial (at 4M), P6 ethanol‐injected mice performed significantly worse in comparison to the saline control—they made more errors and explored the opposite side of the maze more frequently. The ethanol‐treated mice also employed a different search strategy. These results indicate that acute ethanol exposure in early postnatal mice results in specific deficits in long‐term spatial memory retrieval.

It is well known that ethanol exposure can affect memory—Early postnatal acute ethanol exposure leads to significant impairments in long‐term memory in mice. Previous research describes the early postnatal period as a developmental window important for spatial development; P7‐exposed mice did not investigate a novel moved object as readily as saline‐injected animals at 15 weeks of age, though no differences were found in E8‐exposed mice (Sadrian, Lopez‐Guzman, Wilson, & Saito, 2014). P7‐exposure impacted the ability of male mice to learn the Morris Water maze at 2.5M, yet when retested at 8M, these place learning deficits disappeared with age. P7‐exposed mice also learned the working memory version (win‐shift spatial discrimination) of the radial arm maze more slowly when tested at 4.5M (Wozniak et al., 2004).

The ability of our P6 ethanol exposed animals to successfully learn the Barnes maze at 1M may reflect a difference in the particular learning processes required by Barnes maze learning. Successful completion of the Barnes maze taps into hippocampally regulated spatial memory, and our P6 ethanol‐injected animals are able to use these processes successfully at 1M. But early postnatal exposure of ethanol can decrease adult neurogenesis in the dentate gyrus (Sadrian et al., 2014), and this may be related to why we do not see a retention of the Barnes maze information at 4M (early adulthood). An exploration of the effect of impaired cell proliferation due to developmental ethanol on adult learning is warranted.

The Barnes maze also requires striatum‐based implicit learning, since the mice learn to associate the escape tunnel with the removal of noxious stimuli (ultrasonic noise, bright lights, and an open field). In particular, the dorsal striatum is important for the consolidation of memory learning that accrues over many trials (Packard, Hirsch, & White, 1998). As such, future avenues of research should explore how developmental ethanol exposure in the early postnatal period can damage the retrieval processes that occur in the hippocampus and cortex, but should also focus on possible molecular mechanisms behind synaptogenesis and consolidation of long‐term memory in the striatum. Based on our results, we maintain that even a single intoxication event during development will prove detrimental to the long‐term neurological functioning of the fetus.

## Conflict of Interest

None declared.
